# Chloral

**Published:** 1869-11

**Authors:** 


### Chloral.

Dr. Richardson recently opened his course of lectures on Experimental and Practical Medicine. The subject was Chloral; and many new experimental facts were illustrated; among others, the great decrease of animal temperature caused by the substance, and the production of prolonged anæsthesia by inhalation from an ethereal solution. The following is a summary of the lecturer's views given in the *British Medical Journal:* 1. Deep and prolonged narcotism can be safely produced by the hydrate of chloral. 2. During a portion of the period of narcotism, there may be complete anæsthesia with absence of reflex actions; a condition, in short, in which every kind of operation fails to call forth consciousness. 3. During the narcotism, there are intervals of apparent exalted sensibility. 4. In the transition from drowsiness to stupor, there is no stage of muscular excitement; but in birds there is vomiting, as is common in the same animal in the second stage of narcotism from chloroform. 5. During the narcotism produced by the substance, there is invariably reduction of temperature. 6. The hydrate produces muscular relaxation; which relaxation extends to the muscles of volition, and also to the iris and muscular arterial system. From the condition of the muscles after death, it may be inferred that this paralysis is in part due to change within the muscular structure itself. 7. The action of the substance on the nervous system is primarily on the sympathetic ganglia, afterwards on the cerebrum; and, finally, on the heart. 8. Recovery is followed by no bad results. 9. In fatal cases, the functions are destroyed in the following order: *a.* the cerebal; *b.* the voluntary muscular; *c.* the respiratory; *d.* the heart. 10. The substance, in small proportions, prevents, in some degree, the coagulability of the blood; and, in large quantities, stops the process of coagulation altogether. In large quantities, it also destroys the blood-corpuscles, and produces general destruction of blood. But to produce deep insensibility, the dose administered need not be so large as to lead to serious derangement of blood. 11. The phenomena observed correspond with those observed under chloroform; and the balance of evidence is, that they are the result of the action of chloroform. 12. Therapeutically, the agent is to be accepted as the rival of opium. It promises to be useful in cases where there are increment of animal heat, muscular spasm and pain. It will be worthy of extensive trial, in tetanus especially. The dose of hydrate of chloral for a child is seven grains; for an adult, the dose may be extended to one hundred or even one hundred and twenty grains. The lecture was illustrated throughout by experiment; and the lecturer received hearty applause from those who were present.—*Phila. Reporter.*

